# A Commentary on Dual Orphan Nuclear Receptor 4A1 (NR4A1) and NR4A2 Ligands

**DOI:** 10.33696/immunology.8.251

**Published:** 2026

**Authors:** Stephen Safe, James J. Cai, Robert S. Chapkin

**Affiliations:** 1 Department of Veterinary Physiology and Pharmacology, Texas A&M University, College Station, TX 77843, USA; 2 Department of Veterinary Integrative Biosciences, Texas A&M University, College Station, TX 77843, USA; 3 Department of Nutrition, Texas A&M University, College Station, TX 77843, USA; 4 Single Cell Data Science Core, Texas A&M Regional Center of Excellence in Cancer, College Station, TX 77843, USA

**Keywords:** NR4A1, NR4A2, Dual receptor ligands, Inverse agonists, Cancer, Endometriosis, T cell exhaustion

## Abstract

1,1-Bis(3’-indolyl)-1-(3,5-disubstitutedphenyl)methane (DIM-3,5) compounds in the presence or absence of a 4-hydroxylphenyl group bind both orphan nuclear receptor 4A1 (NR4A1) and NR4A2. In cancer cells, these compounds bind and inactivate pro-oncogenic NR4A1 and NR4A2 and downstream pathways acting as inverse agonists that inhibit cancer cell growth, survival, migration and invasion, and induce ferroptosis. Similar results are observed in endometriotic cells where the DIM-3,5 dual NR4A1/2 ligands inhibit NR4A1/NR4A2-mediated pro-endometriotic genes and pathways. The potency of these DIM-3,5 dual NR4A1/NR4A2 ligands is also observed in tumor infiltrating lymphocytes where both receptors are expressed and regulate comparable functions.

## Introduction

**Figure 1.**
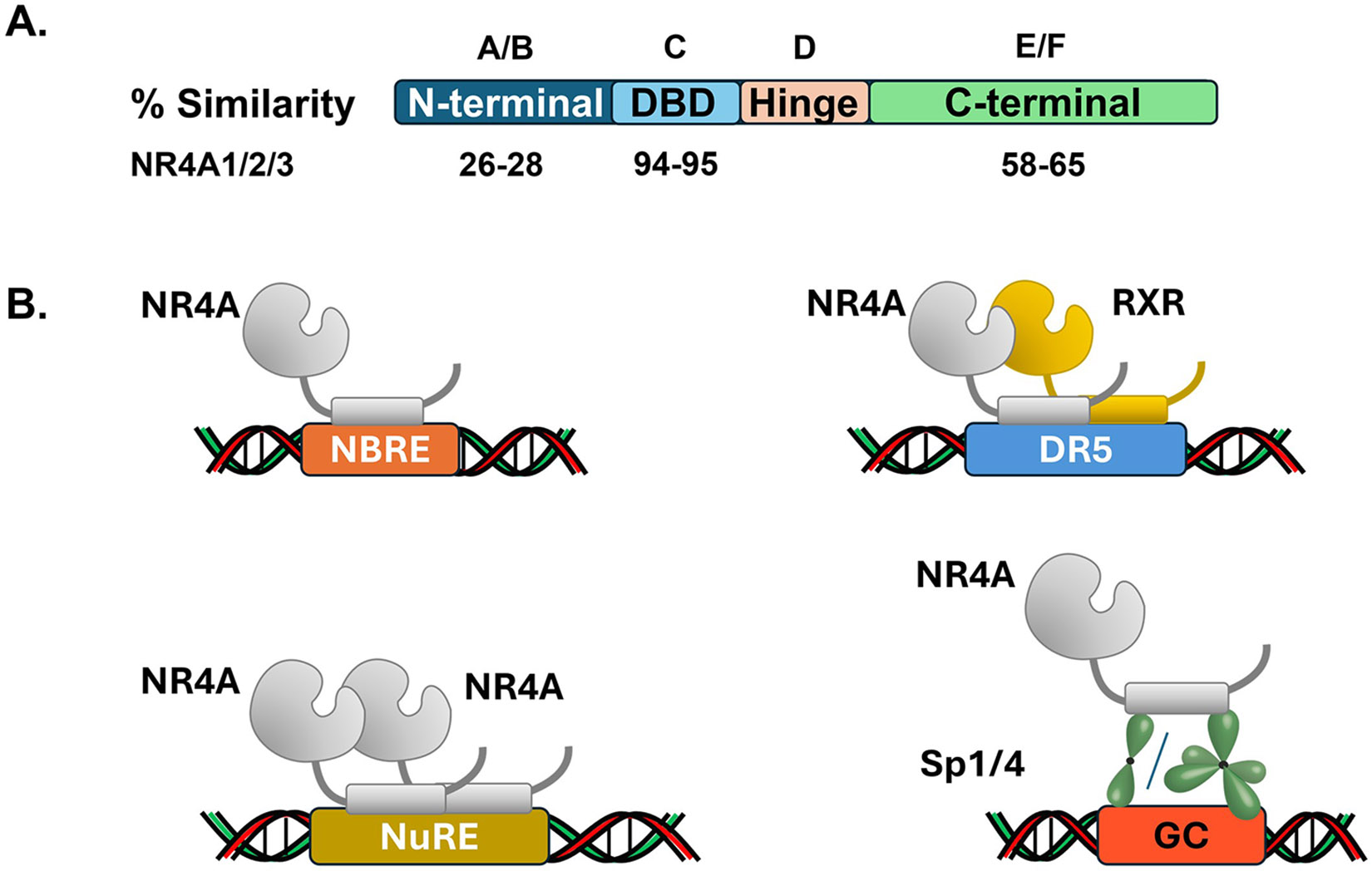
Modular structures of NR4A and transcriptional activation. The domain structure of NRs include the N-terminal (NTD) (A/B), DNA binding (DBD) (C), hinge (D) and C-terminal (CTD) (E/F) domains. The sequence similarities of NR4A1, NR4A2 and NR4A3 are indicated. **B.** NR4A-dependent transcriptional activation involves interaction of NR4A1, NR4A2 or NR4A3 as monomers with an NBRE, as heterodimers with RXR bound to a DRE motif as heterodimers bound to a NuRE or by acting as a cofactor of Sp1 or Sp4 bound to a GC-rich promoter sequence.

There are 48 members of the nuclear receptor (NR) superfamily of transcription factors all of which exhibit a similar modular structure containing a C-terminal ligand binding domain (LBD) and activation function-1 (AF-2), a hinge region, a DNA binding domain (DBD), and an N-terminal domain containing AF-1 [1-3] (Figure 1A). The receptors exhibit amino acid sequence similarities in their DBD and LBDs, but their N-terminal regions are different. The receptors have been classified according to their structural similarities and their endogenous ligands [2] and for 12 of these nuclear receptors endogenous ligands have not yet been identified, and they are classified as orphan receptors. While some orphan receptors may bind endogenous biochemicals, their binding affinities are usually low, and it is unlikely that these compounds are endogenous ligands. Nuclear receptors such as the steroid hormone receptors have been extensively investigated and their roles in maintaining cellular homeostasis and in various diseases have been extensively characterized [1,4]. Moreover, selective receptor modulators (SRMs) have been developed for most NRs, and these compounds exhibit cell context-dependent agonist, inverse agonist or antagonist activities. One example are the selective estrogen receptor modulators (SERMs), such as tamoxifen, that have been extensively used clinically as ER antagonists for treatment of ER-expressing mammary tumors [5-7]. Although endogenous ligands for orphan nuclear receptors have not been identified, evidence for the important roles for these receptors in maintaining cellular homeostasis in both non-cancer- and cancer-related diseases is increasing [1,8,9]. Moreover, development of selective nuclear orphan receptor modulators (SNORMs) for treatment of multiple diseases is ongoing and several promising new therapeutics have been identified [10-12].

## NR4A Subfamily of Orphan Receptors

The NR4A subfamily or orphan receptors includes NR4A1 (Nur77, TR3), NR4A2 (Nurr1) and NR4A3 (Nor1) and they exhibit high amino acid similarities in their DBDs and LBDs (Figure 1A). These receptors were initially identified as immediate early genes induced by nerve growth factor in PC12 cells [13] and have been characterized as genes induced by diverse stimuli/stressors [14,15]. The NR4A subfamily of genes exhibit tissue-specific and overlapping expression and functional activities. It is now clear from cell culture and rodent NR4A knockdown or overexpression that these receptors also display ligand-independent and independent activities. For example, NR4A2 is pro-oncogenic in prostate cancer cells and promotes epithelial-to-mesenchymal transition (EMT) and this is due, in part, to regulation of the Wnt/β-catenin pathway [16]. In prostate cancer cells overexpression of NR4A2 enhances β-catenin expression whereas knockdown of NR4A2 decreases β-catenin levels. The transcriptional activity of NR4A subfamily member includes their binding to specific cis-elements to form monomers, homodimers, and heterodimers with RXR. Recent studies also show that NR4A1 and several other nuclear receptors act as cofactors of specificity protein 1 (Sp1) and Sp4 and possibly Sp3 [17]. NR4A subfamily members alone modulate gene expression through direct interactions with various cis-elements or Sp proteins as illustrated in Figure 1B, and like other NRs their ligands act as agonists or inverse agonists to induce or repress transcriptional activities, respectively.

## NR4A Ligands

**Figure 2.**
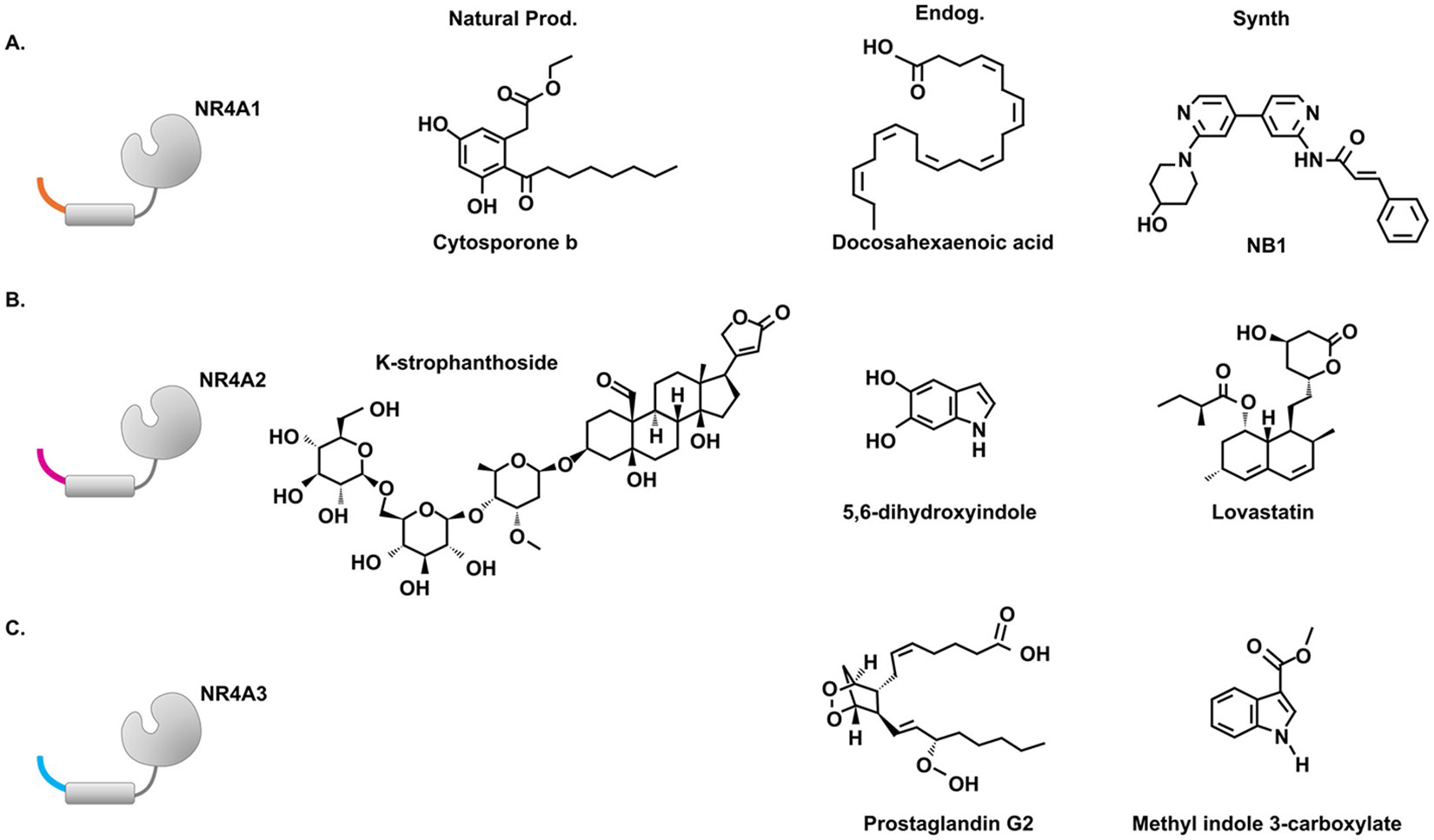
NR4A Ligands. Examples of natural products, endogenous ligands, and synthetic ligands that bind NR4A1 **(A)**, NR4A2 **(B)** or NR4A3 **(C).**

Although initial X-ray crystallographic analysis of NR4A2 showed that bulky amino acid side chains occupy the LBD [18], there has been increasing interest in the structural diversity and function of ligands that bind NR4A1, NR4A2, and NR4A3. Most receptor-ligand binding studies have focused on compounds that interact with NR4A1 and NR4A2 and these include natural products/dietary compounds and derivatives, endogenous biochemicals and synthetic chemicals, and those derived from library screening [19-23]. The natural product, cytosporone B (CsnB), and several structurally related compounds were identified as among the first NR4A1 ligands and extensive studies show that CsnB enhances NR4A1-dependent protection against tissue/organ damage [23-25] (Figure 2). In addition, unsaturated fatty acids such as docosohexaenoic acid [24,26] and synthetic/library derived compounds such as NB1 also bind and activate NR4A1 [27] (Figure 2). There have been multiple studies on the identification and validation of ligands that bind NR4A2 and provide protection from various neurotoxic endpoints where neuronal NR4A2 is an important drug target [28,29]. For example, K-strophanthoside [28] is a naturally occurring NR4A2 ligand. In addition, the dopamine metabolite 5,6-dihydroxyindole [30,31] binds NR4A2 and screening assays have identified synthetic statin drugs as ligands that bind NR4A2 [32]. In contrast, relatively few NR4A3 analogs have been identified, however prostaglandin G2 [33] and methyl indole-3-carboxylate and several other compounds detected by screening a drug fragment library have been identified as NR4A3 ligands [34].

## 1,1-Bis(3’-indolyl)-1-(3,5-disubstitutedphenyl) methane (DIM-3,5) Compounds as NR4A1 Ligands

**Figure 3.**
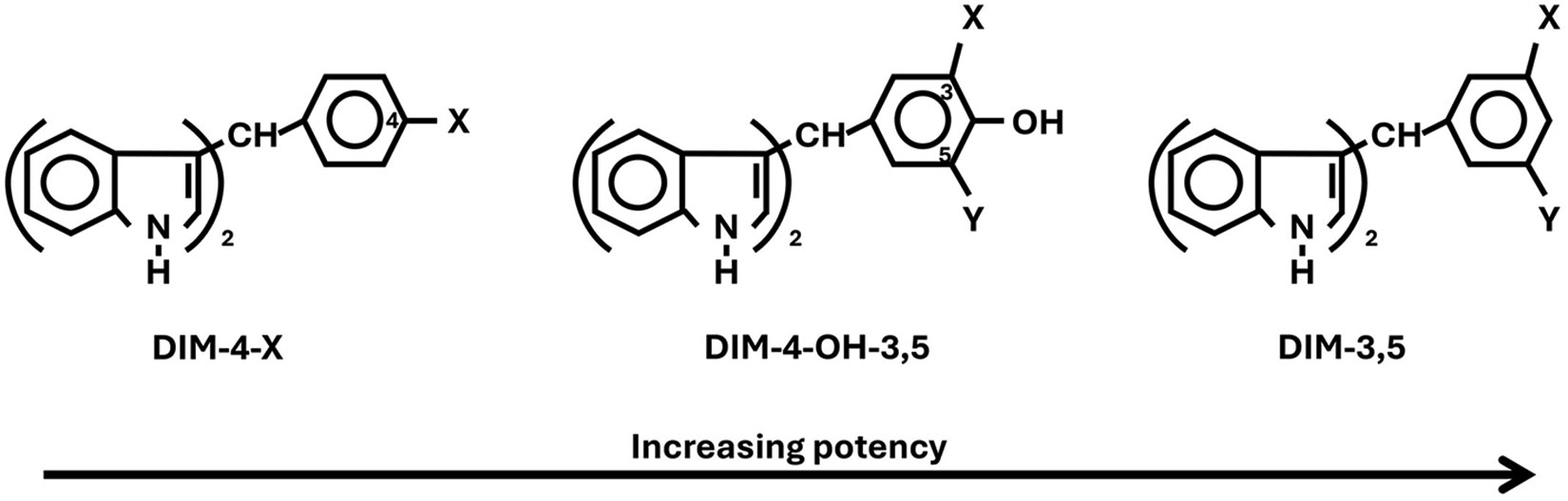
DIM-3,5 Ligands. DIM-4-X, DIM-4-OH-3,5, and DIM-3,5 represent first, second, and third generation NR4A1 ligands which exhibit increasing potency. DIM-4-OH-3,5 and DIM-3,5 compounds bind both NR4A1 and NR4A2 [45].

Initial studies in our laboratory screened a series of 1,1-bis(3’-indolyl)-1-(4-substitutedphenyl)methane (DIM-4) analogs using a fluorescence quenching binding assay. We identified several of these analogs as NR4A1 ligands including those containing a 4-hydroxyphenyl (DIM-4-OH) and 4-carboxymethylphenyl (DIM-4-CO_2_Me) groups [35]. Both of these compounds have been used in several laboratories as NR4A1 ligands and our studies on their *in vivo* activities as inhibitors of solid tumors in mouse models showed that these compounds were active in the 20–50 mg/kg/day range [36,37]. Pharmacokinetic studies showed that serum levels of DIM-4-OH in mice were low and transient due to rapid conjugation of the hydroxyl group [38] and, therefore, a series of 3,5-butressed analogs containing substituents at the 3- and 5-positions of the phenyl group were prepared to inhibit conjugation of the 4-hydroxyl group [39] (Figure 3). Results obtained for the buttressed analogs of DIM-4-OH containing 3-CI, 3,5-Br_2_ and 3-CI-5-OCH_3_ substituents showed that, at doses of 5 mg/kg/day, mammary tumor growth in athymic nude mice bearing orthotopically injected MDA-MB-231 cells was inhibited by > 75% and the IC_50_ for tumor growth inhibition by the 3-CI-5-OCH_3_ analogs was approximately 2 mg/kg/day. At these dose levels, non-specific side effects such as body weight loss, were not observed in the mice treated. A second study investigated the relative potencies of a series of DIM-3,5 analogs which did not contain the 4-hydroxyl group. Interestingly, all of these compounds bound NR4A1, demonstrating that the 4-hydroxyl was not necessary for binding NR4A1 [40]. *In vivo* studies showed that a dose of 1 mg/kg/day DIM-3,5 analogs containing 3-Br-5-OCH_3_, 3-CI-5-OCF_3_ and 3-CI-5-CF_3_ substituents on the phenyl ring significantly inhibited tumor growth (> 60%) in the mouse mammary tumor model [40]. It is assumed that the increased activity of the DIM-3,5 compounds was primarily due to their decreased metabolism and enhanced serum levels, however this has not yet been determined. Subsequent studies have shown that the DIM-3,5 analogs were also potent inhibitors of colon cancer cell and tumor growth due to direct effects on the tumor and enhanced immune surveillance by reversing T cell exhaustion; their anti-endometriosis activity was also observed in endometriotic cells in culture and *in vivo* [41-43]. A potential clinical application for DIM-3,5 ligands would be treatment of cells/tissues expressing mutant NR4As however, cell culture or *in vivo* models for these studies are lacking.

Previous studies in many solid tumor-derived cells and *in vivo* reported that both NR4A1 and NR4A2 were pro-oncogenic and in the initial studies with DIM-3,5 ligands, and DIM-4-CI, these compounds acted as NR4A1 and NR4A2 inverse agonists, respectively [44]. Since the LBDs of NR4A1 and NR4A2 exhibit high amino acid sequence similarity (Figure 1A), another possible mode of action of DIM-3,5 analogs could be their binding to both NR4A1 and NR4A2, thereby simultaneously inhibiting the pro-oncogenic functions of both receptors. Analysis of the DIM-3,5 analogs and the corresponding 4-hydroxyl DIM-3,5 compounds showed that 20 different compounds with variable 3- and 5-substituents bound both NR4A1 and NR4A2 [45]. The K_D_ values were variable and dependent on the type of binding assay and their relative binding affinities for NR4A1 vs NR4A2 were structure-dependent. Using the fluorescence quenching assay, it was evident that compounds that differed in the presence or absence of the 4-hydroxyl group exhibited similar K_D_ values for both receptors and, among the 20 compounds analyzed, the K_D_ values for NR4A1 and NR4A2 differed by less than 2-fold for 12 compounds. K_D_ values for most of these ligands were lower for NR4A1 than NR4A2, however for DIM-3-CI-5-OCH_3_ the K_D_ values for NR4A1 and NR4A2 were 60.3 and 5.2 μM, respectively [45].

**Figure 4.**
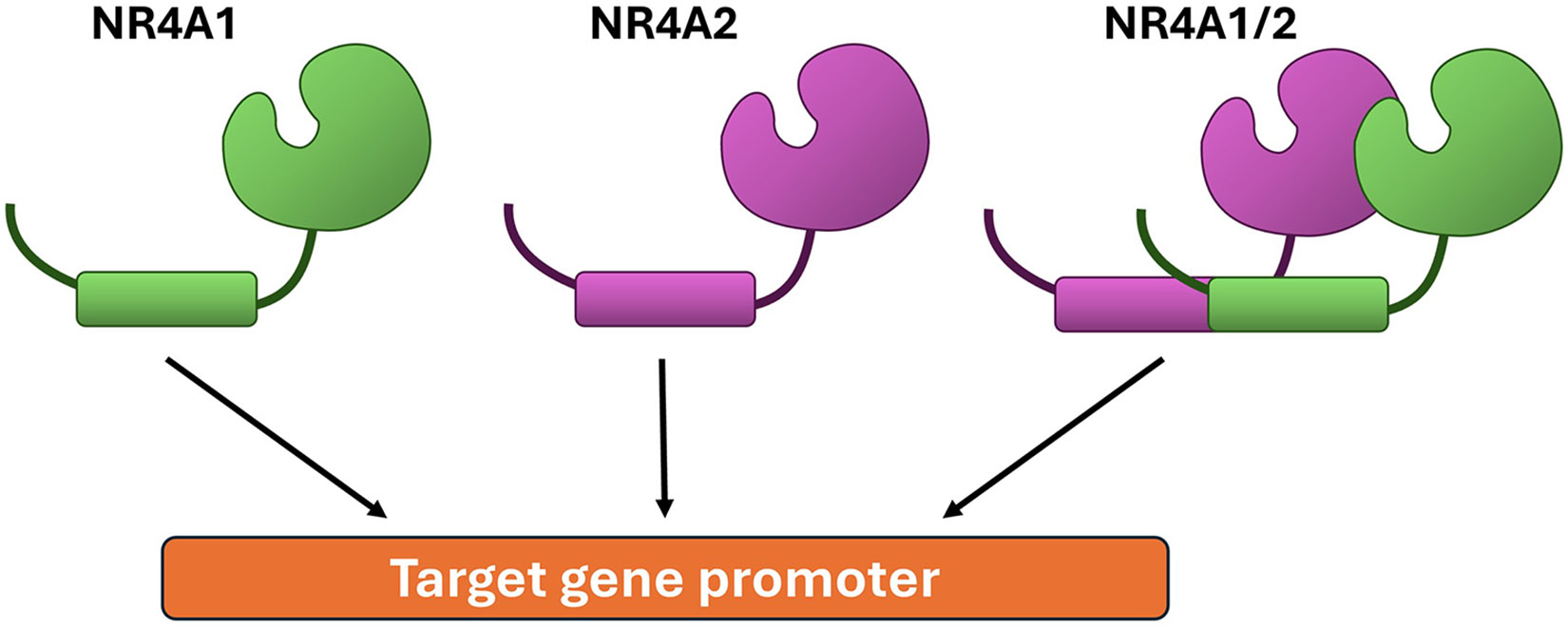
NR4A interaction with target gene promoters. Although both NR4A1 and NR4A2 interact with similar regions of target gene promoters, they may bind as monomers or homo/heterodimers.

The identification of individual compounds that bind more than one NR4A sub-family member is not unique for DIM-3,5 analogs. For example, PGA2 binds NR4A1, NR4A2 and NR4A3 [33,46,47]; there is also evidence that CsnB not only binds NR4A1 but also NR4A2 and NR4A3 [25]. Other studies show that structurally diverse chemicals exhibit binding or transcriptional activation/inhibition of more than one NR4A subfamily member [19,20]. We have also carried out in-depth studies on the effects and mechanisms of action of dual NR4A1/NR4A2 ligands (DIM-3,5) in cancer and in endometriotic cell lines. In colon cancer cell lines, DIM-3,5 analogs downregulated expression of both G9a and β1-integrin, and based on ChIP analysis and other assays, the results suggest that both NR4A1/NR4A2 act as cofactors to enhance Sp1/Sp4-mediated gene expression [48]. Since both NR4A1 and NR4A2 are detected in the “active” promoter regions of G9a and β1-intergin, it is possible that they may act as monomers or homo/heterodimers since it has been reported that NR4A1 and NR4A2 interact [49] (Figure 4). Similar results were observed for NR4A1/NR4A2:Sp1/4 regulation of TWIST1 in glioblastoma, and in breast cancer cells, the transferrin receptor (CD71) was also coregulated by NR4A1/NR4A2:Sp1/4. Although G9a, TWIST1, β1-integrin and CD71 were all coregulated by NR4A1 and NR4A2, DIM-3,5 ligands acted as inverse agonists to decrease expression of G9a, β1-integrin and TWIST1 and as agonist to induce expression of CD71 [50,51]. The gene specific switch in the inverse agonist vs agonist activity of DIM-3,5 compounds demonstrate their selective NR4A modulator activities by altering the expression of genes to inhibit the pro-oncogenic activities of NR4A1 and NR4A2, e.g., induction of CD71 enhanced ferroptosis in breast cancer cells. A similar analysis was carried out in endometriotic cells in which both NR4A1 and NR4A2 play a role in progression of this painful and highly prevalent disease. In this study, the possible role of NR4A1 and NR4A2 (but not Sp1/4) and effects of DIM-3,5 ligands were investigated in both epithelial and stromal cells on expression of pro-endometriotic genes/pathways [e.g., mTOR, fibrosis and epithelial to mesenchymal transition (EMT) genes] [52]. DIM-3,5 treatment and NR4A1/NR4A2 knockdown differentially regulated multiple genes in both epithelial and stromal cells. The results showed that TWIST1, cadherin, ZEB1, Z01, and β-catenin were coregulated by NR4A1 and NR4A2 in both epithelial and stomal derived cells; slug expression was regulated primarily by NR4A1, and all of these genes were downregulated by DIM-3,5 ligands acting as inverse agonists. In contrast, claudin1 was induced after knockdown of NR4A1 and NR4A2 and was also induced by DIM-3,5 acting as an agonist. For all of these genes the effects of DIM-3,5 on gene expression were consistent with their inhibition of EMT and enhancement of the epithelial genotype.

## Summary

The overall results obtained for DIM-3,5 dual NR4A1/NR4A2 ligands in cancer cells are consistent with the common functional effects observed after treatment with individual ligands and after knockdown of NR4A1 or NR4A2. Moreover, chromatin immunoprecipitation analysis shows that both receptors and Sp1/4 colocalize in the “active” GC rich regions of the target gene promoters [48]. However, single cell RNA-seq analysis of the differentially expressed genes in SW480 colon cancer treated with DIM-3,5-CI_2_ and after knockdown of NR4A1/NR4A2 (combined) showed only a modest overlap of commonly induced and repressed genes [48]. These results were obtained after treatment with DIM-3,5-CI_2_ for 24 hours and 72 hours after initiating NR4A1 and NR4A2 knockdown with small inhibitory RNAs. Comparison of differentially expressed genes (DEGs) at these late time points may be problematic and need to be repeated. Despite the limited overlap of DEGs, pathway analysis of the treatment groups converged on several common gene ontology terms and pathways. Thus, in cancer cells the DIM-3,5 dual NR4A1/2 ligands are potent inhibitors of NR4A1- and NR4A2-regulated pro-oncogenic pathways and genes, and their potency is due, in part, to simultaneously targeting both pathways. It was also apparent that many of the pro-oncogenic genes are coregulated by both receptors acting as co-factors of DNA-bound Sp1 and Sp4; however, their interactions with Sp transcription factors as monomers, dimers or heterodimers has not been determined. Since the sequence homology of NR4A1, NR4A2, and NR4A3 ligand binding domains are similar (Figure 1), it is possible that a subset of DIM-3,5 compounds and other previously identified NR4A1 or NR4A2 ligands may also bind NR4A3 which may or may not have functions/activity similar to NR4A1 and NR4A2. This is the subject of an ongoing investigation in our lab.
